# Do we have enough evidences that make you safe to treat a man with hypogonadism one year after a radical prostatectomy for prostate cancer? | *Opinion: Not Yet*

**DOI:** 10.1590/S1677-5538.IBJU.2018.0004

**Published:** 2018

**Authors:** Marcelo Langer Wroclawski, Flavio Lobo Heldwein

**Affiliations:** 1 Hospital Israelita Albert Einstein, São Paulo, SP, Brasil; 2 Faculdade de Medicina do ABC, Santo André, SP, Brasil; 3 Universidade do Sul de Santa Catarina, SC, Brasil; 4 Universidade Federal de Santa Catarina, SC, Brasil

**Keywords:** Prostatic Neoplasms, Hypogonadism, Prostatectomy, Therapeutics

Prostate cancer (PCa) is a heterogeneous disease. After almost a decade of contradictory screening recommendations made by expert and advisory panels (1), prostate cancer has risen again as the second leading cause of cancer death in American males (2).

PCa is androgen dependent. Research on biological effects of testosterone and its relationship with PCa awarded Butenandt with the Nobel Prize in Chemistry in 1939, and Huggins with The Nobel Prize in Physiology or Medicine in 1966 (3, 4). In 1941, Huggins and Hodges observed that castration could cause a decrease of PCa serum marker activity and that administration of exogenous testosterone propionate resulted in its increase. Indeed, different research groups repeatedly showed that PCa culture cells are stimulated by administration of testosterone and that deprivation induces apoptosis (5, 6). These traditional assumptions are the base of metastatic PCa treatment until nowadays.

Similarly to prostate cancer, testosterone-deficiency (TD) (previously named late-onset hypogonadism) prevalence increases with aging. Traditionally an underreported condition, the growing interest on testosterone deficiency resulted in an increase in screening of symptomatic middle-age men and subsequently an exponential boost in testosterone prescriptions, up to 4-fold in the last two decades (7-9).

The main goals of testosterone supplementation therapy (TST) are: restore libido, sexual function, cognition, wellbeing, mood and behavior. Thus, in an evidence-based era, aiming to recommend a safe and effective treatment, we should primarily address the following question: What are the evidences to treat any man with age-related hypogonadism?

A gradual annual decline of testosterone production is related with male aging. If low-testosterone is functional along with a normal aging process or, contrarily, it represents a disease pathologically associated with certain medical conditions such as obesity, smoking, stress and metabolic syndrome, is debatable (10-13). However, TD is commonly used to justify testosterone-replacement, particularly in males with a range of unspecific signs and symptoms, generally associated with aging and/or habits.

A literature review regarding TST on age-related low testosterone could be confusing. Recent guidelines and drug safe communications made by US Food and Drug Administration (FDA) and Australian Endocrine Society have raised skepticism concerning TST (14, 15). Different definitions of biochemical threshold and methods of assessment, lack of consensus on screening and follow-up recommendations, use of different tools to report outcomes even in the same study, low rate of detail reports of study withdrawal, harms and benefits, make medical advice in this field a challenging task.

Another classic example of the controversies that surround testosterone-replacement therapy is its real efficacy. If you are against TST, you are a fan of the recently published systematic review of Huo et al. After reviewing 156 randomized clinical trials, their paper cast doubt on the validity of “low-testosterone” selection of aging male patients who receive TST (16). Even sexual function domains, such as erectile dysfunction and libido, did not find consistent improvements with TST when compared to placebo. The authors concluded that TST is not supported by randomized clinical data. But if you are a TST believer, you will find a solid ground in an abundant amount of papers, such as the RHYME study (17-19). Despite a significant improvement in sexual function measured by IIEF5 questionnaire, the authors acknowledge that this improvement was small, and may have been biased by 25% of the patients reporting a concomitant use of 5 phosphodiesterase inhibitors (17).

Furthermore, recently published reviews and meta-analysis provide support to the hypothesis that exogenous testosterone increases cardiovascular risks (as high as 54% increase of CV events) (20), while others concluded that there is no effect (21-24).

Additionally, since RCTs are usually designed to demonstrate benefit or not with a treatment, adequately powered clinical trials for rare outcomes are challenging. Onasanya et al. reviewed the 6 systematic reviews available on TST and suggested that it would require more than 17,000 participants in each trial group to draw a conclusion about more unusual risks (24). The RCTs published to date varied in size ranging from 29 to 406 men (25). Therefore, we should keep in mind that the majority of prospective studies are underpowered to give a categorical answer about uncommon harms. Also, RCTs often include biased populations enrolled using optimized selection criteria, and those inclusion criteria could not reflect our daily practice.

Despite the popularity and initial enthusiasm with the use of testosterone, several authors report a high discontinuation rate (80-85% after one year) (26). Other critical issue on testosterone-replacement is sponsored trials. In the USA, testosterone therapy sales market rose from an estimated $2 billion in 2012, to $5 billion by 2017 (26). Regarding TST, Alexander et al. reported that the majority of the RCTs (at least 25 of 39) included in their review were sponsored by pharmaceutical companies (25). Thus, data from sponsored trials must be interpreted accordingly, considering a possible lobby of pharmaceutical industry (27, 28).

## Testosterone and prostate cancer

The understanding of the complex biology of prostate cancers is only beginning. However, proponents of TST claim that the growth interest in TST led to a better understanding of the multi-faceted biological relationship between androgens and prostate health and that the conventional wisdom was challenged by the saturation model hypothesis (29). In this model, Morgentaler theorizes that an increased testosterone level above the baseline does not result in further stimulation of prostate cells, because their androgen receptors are fully bound at relatively low serum testosterone concentrations. *In vivo,* the relationship between circulating levels of testosterone and PSA reaches a saturation point approximately at 251 ng/dL (8.7 nmoL/L) (30, 31). However, critics declare that this hypothesis could be simplistic and misleading, not recognizing the different clones of tumor cells that constitute the micro-environment of an aggressive PCa. The unique biology of each clone cells may respond differently to an exogenous stimulus of testosterone, exhibiting different androgen receptors affinity and pathways.

Data regarding levels of serum testosterone and development of PCa are inconclusive. Control arm of the REDUCE trial and population based studies have shown no association between TD and aggressive PCa (32, 33). Nevertheless, several studies point to different conclusions, reporting a direct association between TD and high grade PCa and adverse pathological and clinical features (34-36). In 2005, Teloken et al. reported a relationship between baseline low-testosterone and positive surgical margins (37). PCa genesis occurs in an eugonadic age group, around the 30s and 40s. The above reports, although small and retrospective, are consistent with the natural history of PCa, indicating a reverse association between testosterone and prostate cancer, suggesting that when a PCa develops in a low-testosterone environment, its biological stimulation pathways independent of testosterone make those tumors more aggressive. If this fact proves correct in the future, safety aspects are even more important in TST candidates with PCa history.

The profile of patients undergoing radical prostatectomy has changed. Currently, favorable-risk tumors are been conducted with active surveillance, while intermediate and high-risk PCa are the majority of patients treated with radical surgery (38). The more aggressive the PCa, the more uncertain is its biological behavior.

Mainly expert opinion publications (39, 40) conclude that testosterone replacement is effective and does not raise the risk of biochemical recurrence (BCR) or the need for adjuvant treatments after radical prostatectomy (RP).

Actually, no prospective clinical trials on TST in surgically treated PCa patients have been published. After reviewing the literature, we identified only six small case series (41-46), including a total of 297 cases of PCa patients submitted to RP and post-operatively treated with TST (Table-1).

**Table 1:** Results of testosterone-replacement in men submitted to radical prostatectomy.

	Study type	Total Number of patients	Intermediate and high risk PCa patients	Mean follow- up (months)	BCR	Mortality or metastasis
Kaufman and Graydon, 2004 (41)	retrospective	7	1	24	none	-
Agarwal and Oefelein, 2005 (42)	retrospective	10	8	19	none	-
Khera et al., 2009 (43)	retrospective	57	30	13	none	-
Pastuszak et al., 2013 (44)	retrospective	103	26	27.5	4	-
Kaplan et al., 2014 (45)	retrospective	98*	NA	72	Not mentioned	No risk
Ory et al., 2016 (46)	retrospective	22	19	48	None**	6***

**BCR** = biochemical recurrence; NA – not available;

* = 98 men with >60 days of TST duration.

** = Significant increase in PSA at 50 months (p=0.048)

*** = 6 men received neoadjuvant androgen deprivation

These case series vary in size from 7 to 103 men, as well as follow-up duration from 13 to 72 months. Only 84/297 (29%) had a Gleason ≥7 mentioned. In a retrospective study using Surveillance, Epidemiology, and End Results-Medicare (SEER) database, Kaplan et al. identified 98 post-prostatectomy patients using TST, but no specific stats were provided for those men (45). More recently, Ory et al. reported 22 men treated with RP and subsequently submitted to TST with a mean follow-up of 48 months. No BCRs were registered when adopting the definition of two postop PSA >0.2 μg/L. Nevertheless, the authors noted a statistical significant increase of PSA at 50 months (from undetectable to mean PSA=0.006 μg/L; p=0.048). The authors induced hypogonadism in six men treated with neoadjuvant androgen deprivation prior to RP. Probably, this is an even more specific subpopulation, and should be studied separately (46).

Available data are still far from being either robust or mature. The short-term follow-up data on TST in men that underwent RP does not contribute to understand the downsides of testosterone replacement in this scenario. Also, we need to point out that the risks may have been diluted when we considered low-risk tumors recruited altogether with more aggressive PCas in these case series.

## FINAL CONSIDERATIONS

Low-testosterone could be a normal age-related event or a pathological process associated with certain medical conditions. Contrarily to what proponents of TST believe, neither TST efficacy nor its safety have been undoubtedly proved by an unbiased long-term RCT, even in a healthy population.

There is a complex interaction between testosterone and normal/malignant prostate cells and PCa has distinct androgen stimulation pathways that are still not completely elucidated.

Until nowadays, only results of small observational retrospective studies evaluating TST in patients treated with radical prostatectomy are available and there is no definitive data that the prescription of TST in this scenario is safe and does not lead to BCR or clinical progression, mainly in an era when more aggressive PCa are being treated by surgery.

Discussions on TST should start with what we know regarding benefits and risks of this treatment. Categorical statements about TST safety should be evidence-based. At present, clinicians must inform their patients that oncological outcomes of TST on patients after RP are still unknown.
